# Cardiorespiratory Fitness Is Not Associated with Cardiovascular Disease Risk in Firefighters: A Cross-Sectional Study in South African Firefighters

**DOI:** 10.3390/ijerph21091239

**Published:** 2024-09-19

**Authors:** Tebogo Jenniffer Moselakgomo, Takalani Clearance Muluvhu, Merling Phaswana, Ina Shaw, Brandon S. Shaw

**Affiliations:** 1 Department of Sport, Rehabilitation and Dental Sciences, Tshwane University of Technology, Pretoria 0001, South Africa; tebza.oxtail@gmail.com (T.J.M.); muluvhutc@tut.ac.za (T.C.M.); 2 Department of Exercise Science and Sports Medicine, Faculty of Health Sciences, University of the Witwatersrand, Johannesburg 2193, South Africa; merling.phaswana@wits.ac.za; 3 School of Sport, Rehabilitation and Exercise Sciences, University of Essex, Colchester CO4 3SQ, UK

**Keywords:** aerobic capacity, cardiovascular diseases, cardiovascular health, fire man, maximal oxygen consumption, physical fitness

## Abstract

Cardiovascular disease (CVD) risk factors are frequently reported among firefighters, yet no studies have compared these factors between male and female firefighters, specifically from a low- to middle-income country (LMIC). This study aimed to determine the prevalence of CVD risk factors and their relationship with cardiorespiratory fitness (VO_2max_) in 254 active career firefighters (mean age: 42.6 ± 7.8 years). The assessments included anthropometry, blood pressure, blood glucose, cholesterol, triglycerides, and VO_2max_. The results indicated that 48.0% and 51.8% of females and males were pre-hypertensive, respectively. Hypertension was identified in 15.8% of the firefighters. According to body mass index (BMI), 37.3% of males and 25% of females were found to be overweight, while an additional 44.9% of males and 45.7% of females were classified as obese. Only 17.3% of males and 18.2% of females were found to be of normal weight. These findings were corroborated by categories of central obesity using waist circumference (WC), which were 47.7% for males and 41.6% for females. Low HDL-C was found in 95.2% of males and 86.4% of females, with 28.3% of males also having elevated triglyceride levels (TG). VO_2max_ was “excellent” in 48.8% of males and 12.6% of females, though it had no significant association with most CVD risk factors. The only notable link was a small correlation between VO_2max_ and triglycerides (r = −0.215; *p* = 0.001). These findings suggest that while cardiorespiratory fitness may have no impact, additional factors likely contribute to the cardiovascular health of firefighters, necessitating the need for comprehensive health and fitness programmes.

## 1. Introduction

Firefighters have a wide range of responsibilities that go beyond extinguishing fires. They are also tasked with responding to emergencies, conducting searches and rescues, and protecting health and property in a community [1]. As a result, firefighters experience high physical and psychological stress in their daily work [2]. Firefighting is a physically demanding and dangerous occupation, often requiring maximal physical performance [3]. To meet occupational requirements and ensure safety for themselves, their coworkers, and the public, firefighters must possess a healthy body composition and high levels of flexibility, muscular fitness (including strength and endurance), and cardiorespiratory fitness (VO_2max_) [4]. It is for this reason that the selection process for professional firefighters typically involves evaluating physical fitness, in addition to firefighting experience, to increase the likelihood of success in their chosen profession [5]. 

Due to the physical demands and fitness requirements of their profession, it is often assumed that firefighters are protected from many chronic diseases, such as cardiovascular disease (CVD). As a result of this, several studies have examined the relationship between CVD risk factors and cardiorespiratory fitness (VO_2max_) in firefighters, and such studies generally support an inverse relationship between higher VO_2max_ levels and lower CVD risk. Specifically, a study in 116 male firefighters from upstate New York in the United States of America (USA) found that higher cardiorespiratory fitness in firefighters was strongly linked to improved lipid profiles and lower hypertension, suggesting that improving fitness could reduce CVD risk [6]. Similarly, an inverse relationship was also reported between VO_2max_ and body mass index (BMI), a key CVD risk factor, in 527 male Midwestern career firefighters in the USA with lower fitness levels correlating with higher obesity rates [7]. 

However, other studies have demonstrated that firefighters remain vulnerable to various chronic conditions. Recent epidemiological evidence, for example, indicates a causal link between occupational exposure as a firefighter and certain cancers, such as melanoma and prostate cancer [8,9]. In addition, previous research has indicated that despite their physically demanding jobs, firefighters are at an increased risk for obesity and other CVD risks [10], which can negatively impact their job performance and increase the risk of injury during firefighting activities [11,12]. Specifically, previous research has reported a high prevalence of overweight and obesity among firefighters, with rates ranging from 32% to 40% for obesity and 77% to 90% for combined overweight and obesity [6]. These concerning findings have significant implications for firefighters, as sub-optimal body composition or cardiorespiratory fitness can lead not only to personal injuries but also to risks for colleagues and the public. This is because inadequate physical fitness may impair their ability to respond effectively to emergencies, potentially resulting in substandard performance or sudden incapacitation [4]. 

The unique socio-economic and environmental challenges of firefighters necessitate localised research to tailor preventive strategies and interventions. This is because South African firefighters encounter distinct challenges compared to their counterparts in other regions due to resource limitations, infrastructural issues, and socioeconomic disparities. In this regard, previous research has examined the occupational health challenges faced by South African firefighters and demonstrated how inadequate resources and high levels of occupational stress, exacerbated by socioeconomic constraints, increase risks of injury, stress, and burnout [13]. Similarly, a previous study on cardiorespiratory fitness and cardiovascular health, emphasising the importance of VO_2max_ as an indicator of fitness, established how environmental stressors such as high temperatures and heavy workloads are worsened by resource shortages, which, in turn, affect firefighters’ health [14]. A study by Pillay and van Niekerk [15] explored how socioeconomic factors, such as poor funding, living conditions, and limited access to safety equipment, undermine South African firefighter safety and performance. These studies reveal the pressing need for more targeted research and interventions to address the unique combination of high job demands, resource limitations, and socioeconomic disparities affecting South African firefighters.

This is not a situation unique to South Africa, in that firefighters in many low- and middle-income countries (LMICs) often face distinct challenges compared to those in high-income countries (HICs), including resource constraints, inadequate safety equipment, limited access to health services, and higher exposure to environmental hazards. For instance, a study in Latin American firefighters examined occupational health risks, highlighting issues such as inadequate personal protective equipment (PPE), poor infrastructure, and limited health and safety training, with resource and funding shortages posing major barriers to performance, and health and safety [16]. In Southeast Asia, a study explored the mental health challenges linked to long working hours, low wages, and high exposure to trauma, with a noted lack of mental health support exacerbating these issues [17]. In India, previous research has underscored challenges such as poor working conditions, inadequate safety gear, and toxic fume exposure, calling for improved infrastructure, training, and healthcare access in that region [18]. Similarly, in sub-Saharan Africa, outdated equipment, insufficient training, and limited access to basic health services, compounded by harsh environmental conditions, were demonstrated to significantly strain firefighters [19]. Collectively, these studies underscore the pressing need for targeted interventions and greater investment in firefighter support systems in LMICs to address the significant health, safety, and performance challenges they face. 

Such findings, while localised, can also contribute to global occupational health knowledge, especially in LMICs guide resource allocation, and support evidence-based policies to improve the health and safety of firefighters, thereby benefiting public health and reducing healthcare costs associated with CVD. Therefore, this study aimed to determine the prevalence of CVD risk factors and their relationship with cardiorespiratory fitness in firefighters.

## 2. Materials and Methods

### 2.1. Study Design and Participants

This cross-sectional study included a convenient sample of 254 male and female firefighters in the City of Tshwane Municipality, Gauteng Province, South Africa. In total, 719 firefighters were employed in City of Tshwane Municipality, and using Slovin’s formula and a margin of error of 5%, the representative sample size was found to be approximately 257 participants. To be included in the study, participants had to be employed, active firefighters, be employed by the City of Tshwane Fire Brigade, and be an adult male or female. Permission to conduct the study was granted by the Faculty Committee for Research Ethics, Faculty of Science, Tshwane University of Technology (REC/2022/04/021). Prior to participation, all participants provided informed consent. This study complied with the tenets of the Declaration of Helsinki as revised in 2013.

### 2.2. Procedures

#### 2.2.1. Kinanthropometry Assessment

Participants were evaluated for body composition as per the International Society for the Advancement of Kinanthropometry (ISAK) guidelines [20]. Barefoot height was measured to the closest 0.1 centimetres (cm) using a portable standard stadiometer (Holstein Limited, Crymych, Dyfed, UK), while barefoot body weight was measured to the nearest 0.5 kg using a digital scale (Dismed, Aventura, FL, USA) wearing minimal clothing [21]. Body mass index (BMI) was calculated by dividing the participants’ body mass (kg) by stature squared (m^2^) and expressed as kilogrammes per square meter (kg·m^−2^). Waist circumference (WC) was measured using a non-distendable tape measure (Lufkin, Cooper Tools of Apex, NC, USA) at the mid-point between the iliac crest and bottom of the ribcage.

#### 2.2.2. Blood Pressure Assessment

Blood pressure was measured according to the standards established by the American College of Sports Medicine [21]. Participants’ seated brachial blood pressure (BP) was measured after a five-minute rest using a using a standard-calibrated automated brachial digital BP analyser (Rossmax, Omron, Modderfontein, South Africa). Duplicate measurements were taken consecutively with ≥1 min between each one. The peripheral systolic blood pressure (SBP) and diastolic blood pressure (DBP) were estimated using the mean of these two consecutive readings.

#### 2.2.3. Blood Glucose and Lipid Assessment

Random (non-fasting) glucose, total cholesterol (TC), high-density lipoprotein cholesterol (HDL-C), low-density lipoprotein cholesterol (LDL-C), and triglyceride (TG) levels were determined using a Cardiocheck^®^ PA Analyzer (Polymer Technology Systems, Inc., Indianapolis, IN, USA) [22,23]. Blood samples were drawn using the fingerprick method and a blood droplet placed immediately on Cardiocheck^®^ reagent strips. Samples were drawn while the participants were in a seated position and analysed immediately. The Cardiocheck^®^ PA Analyzer has an accuracy value of r > 0.9858 and a coefficient of variation between test strips not exceeding 9%.

#### 2.2.4. Cardiorespiratory Fitness Assessment

The Queen’s College Step Test was used to assess VO_2max_ in this assessment. This step test was selected as it simulates job-relevant physical demands by involving rhythmic stepping, which mirrors the sustained, rhythmic activity required for tasks such as climbing stairs or moving quickly, which are common in firefighting [24]. In this test, each participant stepped up and down on the stepper using a four-step cadence “up-up-down-down” for three minutes [25]. Participants stopped immediately upon completion of the test, and their heartbeats were counted for 60 s from the end of the test. VO_2max_ was then estimated using the following formulae:


men: VO_2max_ (mL·kg^−1^·min^−1^)
= 111.33 − (0.42 × heart rate (beats per minute (bpm)) (1)

women: VO_2max_ (mL·kg^−1^·min^−1^) =
65.81 − (0.1847 × heart rate (bpm)) (2)

## 2.3. Statistical Analysis

All data were analysed using Statistical Package for Social Sciences (SPSS), version 27.0, for Windows (SPSS Inc., Chicago, IL, USA). An independent *t*-test for normally distributed data and Pearson Chi-square (χ^2^) for the categorical variables were used to examine significant differences in VO_2max_ and CVD risk factors between males and females and for the categories. Differences in clustered risk factors between low and reasonable VO_2max_ groups were assessed using ANOVA. The Spearman Rho correlation coefficients (r) were calculated to determine the relationship between VO_2max_ and CVD risk factors according to the total number of participants and by sex. The classifications were used to interpret correlation coefficients: <0.10 = a small correlation value of 0.30–0.50 indicated a medium correlation, and ≥0.50 showed a larger correlation. Significance levels were set at *p* ≤ 0.05.

## 3. Results

### 3.1. Participant Demographics

A total of 254 firefighters, with a mean age of 42.6 ± 7.8 years, participated in this study. Then, 166 (65.0%) and 88 (35.0%) of those sampled were male and female, respectively. Of these participants, one participant (0.4%) was in the 18–25 years age group, 39 (15.4%) were in 26–35 years age group, 127 (50.3%) were in the 36–45 years age group, 70 (27.6%) were in the 46–55 years age group, and 17 (6.7%) were 56 years or older.

Mean body weight was found to be 86.9 ± 17.6 kg, BMI was 30.5 ± 6.2 kg·m^−2^, and WC was 98.7 ± 14.8 cm. Mean SBP was 125.1 ± 15.6 millimetres mercury (mmHg), while mean DBP was 82.8 ± 10.9. With regard to serum measures, mean random glucose levels were 6.4 ± 2.3 millimoles per litre (mmol·L^−1^), mean TC was 3.7 ± 1.3 mmol·L^−1^, mean HDL-C was 1.2 ± 0.4 mmol·L^−1^, mean LDL-C was 1.7 ± 1.0 mmol·L^−1^, and mean TG levels were found to be 1.7 ± 1.0 mmol·L^−1^. Finally, mean VO_2max_ was found to be 54.6 ± 15.3 mL·kg^−1^·min^−1^ for the sample.

### 3.2. Prevalence of Obesity as Cardiovascular Disease (CVD) Risk Factor

Table 1 presents the percentage distribution of anthropometric profiles for the total participants by sex. The females were significantly (*p* < 0.05) more obese than males (56.8% vs. 45.5%). Female participants also presented with higher levels of class 1 (n = 20 or 22.7%), class 2 (n = 16 or 18.2%), and class 3 (n = 14 or 15.9%) obesity compared to class 1 (n = 51 or 30.7%), class 2 (n = 19 or 11.4%), and class 3 (n = 6 or 3.6%).

The findings also demonstrated that that females had a higher WC than males but were non-statistically presented with a higher-risk WC classification (n = 42 or 47.7%) compared to their male colleagues with high-risk WC classification (n = 69 or 41.6%). Additionally, females non-significantly showed a very high risk of WC compared to their male counterparts (n = 14 or 15.9% compared to n = 14 or 8.4%, respectively).

### 3.3. Prevalence of Hypertension as Cardiovascular Disease (CVD) Risk Factor

**Figure 1:**
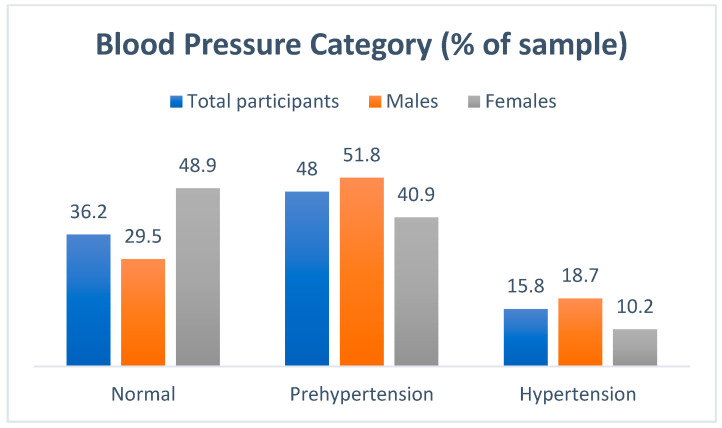
Prevalence of hypertension as cardiovascular disease (CVD) risk factor in South African firefighters.

Of all the firefighters, 36.2% presented with normal blood pressure values, 48.0% were found to be pre-hypertensive, and 15.8% were hypertensive (Figure 1) [27]. Specifically, of the male firefighters, 29.5% presented with normal blood pressure values, 51.8% were found to be pre-hypertensive, and 18.7% were hypertensive. In turn, 48.9% of female firefighters had normal BP values, 40.9% were pre-hypertensive, and 10.2% could be classified as hypertensive.

### 3.4. Prevalence of Hyperglycaemia and Dyslipidaemia as Cardiovascular Disease (CVD) Risk Factor

**Figure 2:**
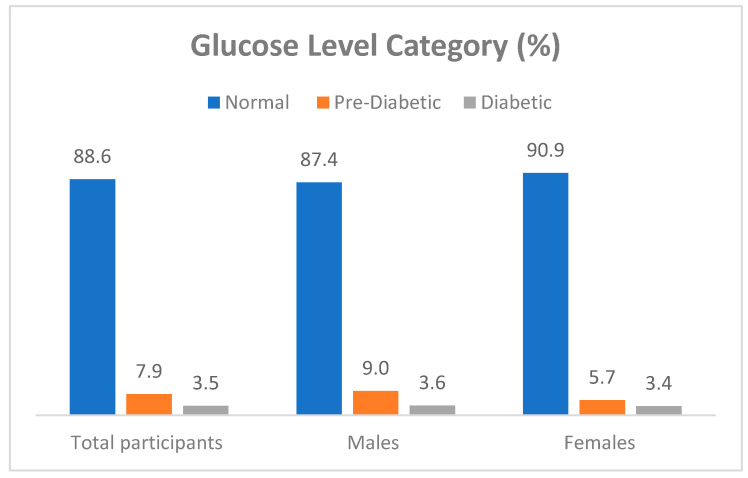
Prevalence of hyperglycaemia as cardiovascular disease (CVD) risk factor in South African firefighters.

The results of this study show that 88.6% of the firefighters had normal blood glucose levels, whereas 7.9% could be classified as pre-diabetic (Figure 2) [26]. Although no participants reported a diagnosis of diabetes in this cohort, 3.6% of males and 3.4% of females had glucose levels that could be indicative of diabetes.

Male firefighters were found to have similar TC levels (*p* = 0.298) and “borderline risk” (9.0% vs. 5.7%, *p* = 0.298) as well as “high-risk” (1.8% vs. 1.1%) from TC levels when compared to the female firefighters (Table 2). From these results, 4.2% of male and 1.1% female firefighters had borderline risk levels of LDL-C (*p* = 0.249). However, the male firefighters had significantly low HDL-C levels when compared to the female firefighters (95.2% vs. 86.4%, respectively, *p* = 0.013). Similarly, they had significantly higher “borderline high” (male: 16.9% vs. female: 18.1%), “high” (male: 28.3% vs. female: 6.8%), and “very high” (male: 1.6% vs. female: 2.4%).

### 3.5. Prevalence of Poor Cardiorespiratory Fitness (VO_2max_) as Cardiovascular Disease (CVD) Risk Factor

**Figure 3:**
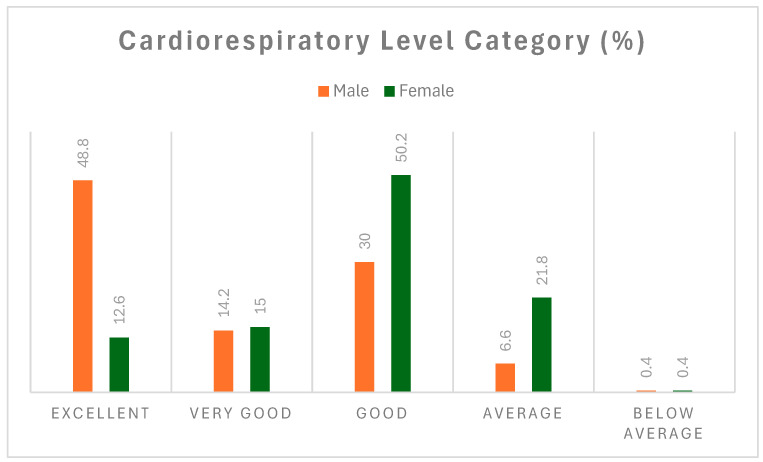
Prevalence of poor cardiorespiratory fitness (VO_2max_) as cardiovascular disease (CVD) risk factor in South African firefighters.

The results of this study showed that 48.8% of male firefighters had “excellent” VO_2max_ levels compared to 12.6% of their female counterparts [27] (Figure 3). In addition, 14.2% of males and 15% of females had “very good” VO_2max_ levels. For the “good” VO_2max_ category, 30% of males and 50.2% of females were included. Meanwhile, 6.6% of males and 21.8% of females had “average” VO_2max_ levels. Finally, 0.4% of both male and female firefighters displayed “below average” VO_2max_ levels.

### 3.6. Correlation Matrix for Cardiorespiratory Fitness (VO_2max_) and Cardiovascular (CVD) Risk

Post hoc testing demonstrated no significant correlations between VO_2max_ and BP (r = −0.83; *p* = 0.189), BMI (r = 0.029; *p* = 0.643), WC (r = −0.014; *p* = 0.819), blood glucose (r = −0.021; *p* = 0.745), TC (r = −0.051; *p* = 0.422), HDL-C (r = 0.102; *p* = 0.105), LDL-C (r = 0.011; *p* = 0.865). Interestingly, VO_2max_ had a small, significant association with TG (r = −0.215; *p* = 0.001).

## 4. Discussion

CVD is the most frequent cause of on-duty mortality among firefighters [6,28] and may be associated with underlying CVD risk factors [29]. Thus, the aim of this study was to determine the prevalence of CVD risk factors and their relationship with cardiorespiratory fitness in firefighters. In this regard, our study revealed a significant prevalence of CVD risk factors among both male and female firefighters. Notably, the present study found that despite the strenuous nature of emergency duty, firefighters had concomitant high VO_2max_ levels and displayed a high prevalence of obesity (through both BMI and WC categorisation) and dyslipidaemia (including low HDL-C, and high TG).

The data from this study indicate alarming rates of overweight and obesity among the participants, with 37.3% of males and 25% of females classified as overweight according to BMI, and a further 44.9% of males and 45.7% of females classified as obese. These findings are concerning given the well-established link between obesity and various CVD risk factors. The high prevalence of central obesity, as indicated by waist circumference, further exacerbates these concerns, suggesting a need for targeted interventions focusing on weight management and the reduction of central adiposity. The epidemic of obesity in society seems to be mirrored in this and other samples of firefighters. In this regard, the prevalence of obesity in firefighters ranges between 12% and 60% [6,10,12,30,31,32,33]. Soteriades et al. [23] also demonstrated that the obese firefighters in their sample were more likely to have hypertension and low HDL-C at follow-up. While studies on CVD risk prevalence are unique for female firefighters, the study of Gendron et al. [32] only demonstrated a 12% prevalence of obesity in their sample of 41 Canadian female firefighters compared to the 45.7% of female firefighters in this study.

Hypertension is a major risk factor for cardiovascular disease (CVD), and screening for it in the general population is one of the most important preventive measures to reduce CVD morbidity and mortality. Previous studies have found hypertension prevalence in 5–44% of firefighters [30,32,34,35,36,37]. Interestingly, the lowest prevalence of hypertension was found in a study utilising a female-only sample of Canadian firefighters [32]. While the finding by Soteriades et al. [37] that 23% of their firefighter sample had hypertension is innocuous, more concerning, however, was that despite 22% being on antihypertensive medication, a significant 74% of hypertensive firefighters were not adequately controlled.

The lipid profile results were particularly striking, with 95.2% of males and 86.4% of females exhibiting low levels of HDL-C, a protective factor against CVD [38]. Additionally, a significant proportion of males (28.3%) had high TG, highlighting the need for dietary and lifestyle interventions aimed at improving lipid profiles. These dyslipidaemic patterns underscore the necessity for regular lipid screening and management in the firefighter population. Previous studies in firefighters confirm this study’s findings and showed dyslipidaemia prevalence rates of between 5% and 56% [6,30,32,39,40]. However, the findings of Gendron et al. [32] refute this study’s findings, as only 5% of their female fighters were found to have dyslipidaemia. In turn, while no participants reported a diagnosis of diabetes, the results of this study demonstrate that 7.87% of the sampled firefighters could be classified as pre-diabetic, and 3.61% of males and 3.41% of females had glucose levels that could be indicative of diabetes. This is consistent with the study of Gendron et al. [32] that reported a type 2 diabetes mellitus prevalence of 3% in their female firefighter cohort.

A key finding of our study is the disparity in cardiorespiratory fitness levels between male and female firefighters, with 48.8% of males having “excellent” VO_2max_ levels compared to only 12.6% of females. Despite this difference, our analysis revealed no significant associations between VO_2max_ and most CVD risk factors, including blood pressure, BMI, waist circumference, blood glucose, TC, HDL-C, and LDL-C. The only notable exception was a small but significant inverse association between VO_2max_ and TG, suggesting that higher VO_2max_ may contribute to lower TG levels.

In South Africa, research on the relationship between VO_2max_ and CVD risk factors in the general population is limited. However, some relevant studies from South Africa and neighbouring regions have addressed related topics. Steyn and Fourie [41] investigated the impact of physical activity on CVD risk factors in South African adults. Their study found that while physical activity, which influences VO_2max_, was associated with certain cardiovascular health indicators, the direct link between VO_2max_ and specific CVD risk factors was mixed. Similarly, Monyeki and Toriola [42] assessed VO_2max_ among South African adolescents and found that although VO_2max_ is crucial for overall health, the association between VO_2max_ and specific CVD risk factors was not consistently significant. In another study, Pienaar and Monyeki [43] explored the relationship between VO_2max_ and CVD risk factors in South African women, observing that the associations varied, and in some cases, significant links were not found. These studies indicate that while VO_2max_ remains an important measure of cardiorespiratory fitness, its direct correlation with specific CVD risk factors can be inconsistent in the South African context, potentially due to variations in population characteristics, lifestyle factors, and methodological differences.

Limited studies have explored the relationship between VO_2max_ and CVD risk factors across various occupations, with some reporting no significant associations. Williams and Egan [44] examined cardiorespiratory fitness among police officers and found that while VO_2max_ generally indicated cardiovascular health, its direct association with specific CVD risk factors was not consistently significant. In turn, Johnson and Smith [45] studied construction workers and observed that while VO_2max_ was linked with general cardiovascular health, its relationship with particular CVD risk factors was not significant in this population. As with the findings of this study, those findings suggest that VO_2max_, while a valuable marker of cardiorespiratory fitness, may not consistently correlate with specific CVD risk factors depending on the occupation and other contextual variables.

Another particularly noteworthy finding of this study is that it appears that obesity is associated with a clustering of CVD risk factors, as with the general population, even in this “fit” population. This contrasts with previous research that indicates that these risk factors consistently lower cardiorespiratory fitness in firefighters [46,47,48,49,50]. Interestingly, when an increased cardiorespiratory fitness has been found, it has been demonstrated to have beneficial effects on CVD risk factor profiles among firefighters, irrespective of BMI [51]. Although previous research has indicated that BMI has independent favourable effects on CVD risk [51], the results of this study may indicate that obesity is equally likely to be associated with CVD risk in firefighters as cardiorespiratory fitness. A previous systematic review by Soteriades et al. [28] found that firefighters had an increased prevalence of low cardiorespiratory fitness, obesity, and other CVD risk factors. This is somewhat in contrast to the present study’s findings that demonstrated high levels of cardiorespiratory fitness in our cohort of firefighters.

This study has several limitations which should be recognised. Although the sample consisted of 254 active firefighters, this number may not be representative of all firefighters globally, or even regionally. Age-related fitness declines were neither assessed nor controlled for. As individuals age, VO_2max_ naturally decreases due to reduced heart rate, muscle loss, and diminished cardiovascular function. This decline can confound the relationship between VO_2max_ and CVD risk factors, as older individuals may have lower VO_2max_ levels independent of their cardiovascular health. No information was collected on the use of anti-hypertensive medications or cholesterol-lowering medications among these study participants and may not provide a complete overview of those already diagnosed with hypertension and diabetes. In addition, blood glucose and lipid profile were based on random/non-fasting blood draws as explained in the methods section, thereby limiting our ability to perform further analyses on other conditions such as impaired fasting glucose. Also, the present study utilised BMI and WC rather than direct measurements of percentage body fat to determine adiposity. While these measures are valid, reliable and provide a basis for comparison across studies, BMI may overestimate overweight and obesity among muscular firefighters, hence the inclusion of WC as an additional anthropometric measure. This study did not evaluate the firefighters’ diets, so we cannot link our obesity findings to this lifestyle factor.

## 5. Conclusions

The findings of this study demonstrate that South African firefighters have increased cardiorespiratory fitness due to the high physical demands and occupational hazards they face. However, contrary to the general population, this higher cardiorespiratory fitness does not mitigate the risk from other CVD risks, especially dyslipidaemia and obesity. Further, our study highlights a significant prevalence of CVD risk factors among both male and female firefighters, with no substantial associations between these risk factors and cardiorespiratory fitness, except for TG. Despite being a crucial factor in determining fitness for duty, obesity is problematic in the firefighting services. This is despite high levels of cardiorespiratory fitness.

Future studies should focus on further exploring the sex-specific differences in CVD risk factors among firefighters, as this study revealed significant disparities between male and female participants. Given the high prevalence of CVD risk factors across both sexes, future research should investigate the underlying causes, including occupational stressors, environmental exposures, and lifestyle factors unique to firefighting. Additionally, while VO_2max_ showed limited correlation with most CVD risk factors, its relationship with other metabolic markers and long-term cardiovascular outcomes warrants deeper investigation. Longitudinal studies are needed to assess how CVD risk factors evolve over time in relation to firefighters’ fitness levels and to develop targeted interventions that address both physical fitness and other health determinants. This will aid in reducing the risk of on-duty cardiovascular events and improving the overall health and safety of this population and those they attend.

This is because firefighters’ physical fitness, including body composition, and not just cardiorespiratory fitness, is vital for not only their own health, but also for public safety. To tackle this issue, in addition to professionally regulated occupation-specific entry physical and skills testing, all fire departments should establish and conduct regular medical and physical fitness assessments. Poor outcomes arising from these assessments, and following any significant illness, should automatically mandate a thorough return-to-work evaluation and presentation of dietary and exercise guidelines. Further, given the observed differences in CVD risk factors and VO_2max_ levels between male and female firefighters, sex-specific interventions may be necessary. Tailoring programmes to address the unique physiological and occupational challenges faced by female firefighters could enhance the effectiveness of these interventions and improve overall cardiovascular health outcomes.

## Figures and Tables

**Table 1:** Anthropometric profiles as cardiovascular disease (CVD) risk in South African firefighters.

		Total (N = 254)	Male (n = 166)	Female (n = 88)	Significance (*p*-Value)
		n (%)	n (%)	n (%)	
BMI (kg·m^−2^)	Normal weight †	44 (17.3)	28 (16.9)	16 (18.2)	0.001 *
	Overweight †	65 (29.5)	62 (37.3)	22 (25.0)	
	Obesity class 1 †	65 (29.5)	51 (30.7)	20 (22.7)	
	Obesity class 2 †	19 (8.6)	19 (11.4)	16 (18.2)	
	Obesity class 3 †	15 (6.8)	6 (3.6)	14 (15.9)	
WC (cm)	Low risk †	12 (4.7)	10 (6.0)	2 (2.3)	0.140
	Low †	103 (40.6)	73 (44.0)	30 (34.1)	
	High risk †	111 (43.7)	69 (41.6)	42 (47.7)	
	Very high risk †	28 (11.0)	14 (8.4)	14 (15.9)	

* Statistical significance between males and females at *p* ≤ 0.05; BMI: Body mass index; WC: Waist circumference; † American College of Sports Medicine (2022) [26].

**Table 2:** Lipid profiles as cardiovascular disease (CVD) risk in South African firefighters.

		Total (N = 254)	Male (n = 166)	Female (n = 88)	Significance (*p*-Value)
		n (%)	n (%)	n (%)	
Total cholesterol (mmol·L^−1^)	Desirable †	230 (90.6)	148 (89.2)	82 (93.2)	0.298
	Borderline High †	20 (7.9)	15 (9.0)	5 (5.7)	
	High †	4 (1.6)	3 (1.8)	1 (1.1)	
HDL-C (mmol·L^−1^)	Low †	234 (92.1)	158 (95.2)	76 (86.4)	0.013 *
	High †	20 (7.9)	8 (4.8)	12 (13.6)	
LDL (mmol·L^−1^)	Optimal †	205 (80.7)	138 (83.1)	67 (76.1)	0.249
	Above Optimal †	40 (15.7)	20 (12.0)	20 (12.0)	
	Borderline High †	8 (3.1)	7 (4.2)	1 (1.1)	
	High †	4 (2.4)	1 (0.4)	-	
TG (mmol·L^−1^)	Normal †	154 (60.6)	85 (51.2)	69 (78.4)	0.001 *
	Borderline high †	43 (16.9)	30 (18.1)	13 (14.8)	
	High †	53 (20.9)	47 (28.3)	6 (6.8)	
	Very high †	4 (1.6)	4 (2.4)	0 (0.0)	

* Statistical significance between males and females at *p* ≤ 0.05; † American College of Sports Medicine (2022) [26]; mmol·L^−1^: millimoles per litre; HDL-C: high-density lipoprotein cholesterol; LDL-C: low-density lipoprotein cholesterol; TG: triglycerides.

## Data Availability

The raw data supporting the conclusions of this article will be made available by the authors on request.
